# Sport participation after isolated distal femoral osteotomy in young patients: High return‐to‐sport rates and predictive role of preoperative activity level

**DOI:** 10.1002/ksa.70524

**Published:** 2026-07-13

**Authors:** Gian Andrea Lucidi, Federico Maria Adravanti, Claudio Rossi, Margherita Bonaiuti, Stefano Di Paolo, Alberto Grassi, Stefano Zaffagnini

**Affiliations:** ^1^ II Clinica Ortopedica e Traumatologica IRCCS Istituto Ortopedico Rizzoli Bologna Italy; ^2^ Dipartimento di Scienze Biomediche e Neuromotorie (DIBINEM) University of Bologna Bologna Italy

**Keywords:** DFO, distal femoral osteotomy, osteotomy, return to sport, return to work

## Abstract

**Purpose:**

The present study aimed to assess return to sport (RTS) rate, activity level and return to work (RTW) in young, physically active patients who underwent isolated distal femoral osteotomy (DFO) for lateral knee pain and valgus malalignment.

**Methods:**

A retrospective analysis was conducted on consecutive isolated DFO procedures performed at a single institution with a minimum 2‐year follow‐up. Inclusion criteria specified patients under the age of 45 who were active in sports 2 years prior to surgery. Data were collected regarding RTS rate, type of sports, sports impact level, RTW and complications. Univariate regression analyses were performed to identify predictors influencing RTS and the level of RTS.

**Results:**

Thirty‐four patients (mean age 31.7 ± 9.7 years) were evaluated at a mean follow‐up of 7.2 ± 3.8 years. The mean preoperative hip–knee–ankle (HKA) was 187.1 ± 3.1 with a mean achieved correction value of 7.7 ± 2.5°. The RTS rate was 79.4% (27/34), with a mean time to resumption of 5.4 ± 5.4 months. Among those returning to sports, 85.2% maintained their pre‐symptom IMPACT level, though no patients reported an improvement in status. At the final follow‐up, 67.7% remained active, averaging 4.5 ± 7.1 h of sports participation per week. Univariate regression identified the pre‐symptom Tegner scale as a significant predictor of absolute RTS (odds ratio = 2.33, *p* = 0.018). Regarding occupational outcomes, 100% of patients returned to work at a mean of 4.4 months, with 86.4% of active workers resuming previous or similar physical demands. The complication rate was 11.8% (4/34), with no clinical failures reported.

**Conclusions:**

DFO performed in a young and active population results in high RTS rates and continuous sports participation even 7 years after surgery. Moreover, higher pre‐symptoms Tegner scale was associated with higher odds of returning to sport compared to lower Tegner scale values.

**Level of Evidence:**

Level IV case series.

**Abbreviations**
BMI: body mass index
DFO: distal femoral osteotomy
HKA: hip–knee–ankle
HTO: high tibial osteotomy
IKDC: International Knee Documentation Committee
KOOS: Knee injury and Osteoarthritis Outcome Score
LDFA: lateral distal femoral angle
LOWDFO: lateral opening wedge distal femoral osteotomy
MCID: minimal clinically important difference
MCWDFO: medial closing wedge distal femoral osteotomy
MPTA: medial proximal tibial angle
MRI: magnetic resonance imaging
NSAIDs: non‐steroidal anti‐inflammatory drugs
OA: osteoarthritis
PROMs: patient‐reported outcome measures
RTS: return to sport
RTW: return to work
SD: standard deviation
TKA: total knee arthroplasty

## INTRODUCTION

Osteotomies around the knee are joint‐preserving surgical techniques aimed at restoring physiological alignment and redistributing load across the knee [26, 30]. These procedures are particularly relevant in young and physically active patients presenting with symptomatic deformity, unilateral compartment overload and early stages of osteoarthritis (OA) [37]. In this population, joint‐preserving strategies are particularly important, as knee arthroplasty procedures may yield inferior long‐term outcomes in younger individuals with higher functional demands and longer life expectancy [14].

Distal femoral osteotomy (DFO) represents the preferred corrective option for valgus malalignment originating primarily from the distal femur [18, 20].

The procedure is most frequently performed in patients with isolated lateral compartment OA or lateral compartment overload, and it can be combined with other knee preservation procedures such as cartilage restoration or meniscal allograft transplantation [11, 20, 32].

Compared with varus‐correcting procedures such as high tibial osteotomy (HTO), valgus‐correcting osteotomies are performed less frequently and the available clinical evidence remains limited [10].

With the increasing use of realignment osteotomies in young and physically active individuals, outcomes related to return to sport (RTS) and return to work (RTW) have become increasingly relevant when evaluating the effectiveness of these joint‐preserving procedures. In highly active patients, the ability to resume sports participation and physically demanding activities represents a key factor influencing both treatment decisions and patient satisfaction.

RTS after HTO has been increasingly investigated in recent years, with several studies reporting high RTS rates and sustained sports participation in active populations. However, comparable data regarding DFO remain limited [12, 17, 21].

Most studies evaluating outcomes after DFO include relatively small cohorts, heterogeneous patient populations or patients undergoing concomitant procedures such as ligamentous reconstruction, cartilage restoration procedures or meniscal transplantation. Consequently, the true rates of RTS and RTW after isolated DFO in young and physically active patients remain poorly defined [7, 9, 24, 29, 34].

Therefore, the purpose of the present study was to evaluate RTS and RTW rates, activity levels and complications in young and physically active patients who underwent isolated DFO for symptomatic valgus malalignment. Additionally, we aimed to investigate potential patient‐related factors associated with RTS in this population. The rationale of this study was to investigate whether DFO in patients with a valgus knee malalignment is associated with high rates of RTS and durable long‐term sports participation. This question is particularly relevant because DFO is still commonly perceived in the literature as an invasive procedure with limited postoperative sports activity. Furthermore, evidence on this topic remains scarce, with only a few studies specifically assessing RTS outcomes following DFO.

## MATERIALS AND METHODS

### Study design and patient selection

A retrospective study was conducted at a single institute (Rizzoli Orthopaedic Institute, Bologna, Italy). Moreover, 358 patients who underwent DFO between April 2004 and May 2023 were screened for eligibility. The study was approved by the Local Ethical Committee of CE/AVEC IOR, Italy (Protocol No. 887/2022/Oss/IOR) and was conducted according to the principles of Helsinki.

Patients under 45 years of age who participated in sports 2 years prior to undergoing surgery and who had symptomatic valgus malalignment without end‐stage OA were included in our study. Inclusion and exclusion criteria were applied to obtain a homogeneous population of active patients (see Table 1 for details).

**Table 1:** Inclusion and exclusion criteria for the present study.

Inclusion and exclusion criteria
Inclusion criteria
Minimum 2 years of follow‐upAge between 18 and 45 yearsIsolated DFO (closing or opening wedge)Kellgren–Lawrence Grade I–IIIParticipation in organized or recreational sport activity at least 2 times a week for at least 2 h/week in the 2 years before DFO
Exclusion criteria
Osteonecrosis in the involved kneeCombined procedure (ACL reconstruction, meniscus transplant, …)Double level osteotomySkeletal immaturityPrevious trauma with osteosynthesis in the ipsilateral kneeKellgren–Lawrence Grade IVPatients not involved in sport activity for the 2 years before surgery

Abbreviations: ACL, anterior cruciate ligament; DFO, distal femoral osteotomy.

The indication for surgery in this cohort was valgus knee malalignment (minimum 4°) with concomitant persistent pain and functional limitations in daily life and sports activities, unresponsive or worsening after at least 6 months of conservative treatment. The latter included cyclic ice, non‐steroidal anti‐inflammatory drugs, physical therapy, corticosteroid and hyaluronic acid injections and varus insoles.

### Patients evaluation

Demographics and surgical data were obtained via hospital databases. All patients were contacted between April 2025 and October 2025, with at least 2 years of follow‐up.

Patients received multiple questions regarding their sport activity: type and level (recreative, competitive or professional) of sport practiced before symptoms, in the preoperative period and at follow‐up; impact level of sports activity (low, intermediate and high) both preoperatively and at follow‐up (Supporting Information S1: Appendix 1); time to RTS after surgery and frequency (hours per week) of sport activity at follow‐up.

Additionally, patients were assessed during study inclusion with the following validated clinical scores, including Lysholm [22], Tampa Scale for Kinesiophobia (TSK) [35], Sports score [4] and the Likert scale [23]. Visual analogue scale (VAS) [5] for pain was assessed preoperatively, after resuming sports (if achieved), and at the time of follow‐up, in order to extensively evaluate the level of pain of the patients after the procedure. Additionally, the Tegner [33] activity score was obtained during study inclusion regarding two different time points: before the onset of knee symptoms and at the last follow‐up. The latter clinical score and Impact activity level were evaluated only before the onset of symptoms and at follow‐up because most patients were not actively participating in sports in the immediate preoperative period due to disabling symptoms limiting the overall quality of life.

Working activity was divided into sedentary and active based on carrying heavy loads, hours of standing and walking distance. Data regarding pre‐ and postoperative working activity (either sedentary or active) and timing to RTW were obtained. Finally, data regarding complications or additional surgeries on the operated knee during the follow‐up period were collected.

### Preoperative planning

All patients were assessed preoperatively with anteroposterior long‐leg weight‐bearing X‐rays to determine the hip–knee–ankle (HKA) angle, the medial proximal tibial angle (MPTA) and the lateral distal femoral angle (LDFA) for the confirmation of the presence of mainly femoral valgus deformity [38]. The preoperative planning was performed on long leg weight‐bearing radiographs according to Marcheggiani et al., both for closing and opening wedge osteotomies [25]. The deformity correction aimed to obtain a neutral mechanical alignment with a postoperative HKA angle of 0°–2° varus, avoiding corrections that could excessively alter the obliquity of the joint rim, considered as a postoperative LDFA higher than 95° [2]. The decision whether to perform a closing wedge (CW) or opening wedge (OW) osteotomy was made based on the eventual preoperative lower limb discrepancy and the surgeon's preference.

### Surgical technique

Diagnostic arthroscopy was performed before the osteotomy only in cases of a probable meniscal lesion detected at magnetic resonance imaging (MRI).

#### Medial closing wedge distal femoral osteotomy (MCW‐DFO)

The patient lies supine with the knee flexed in the ‘figure of four’ position, in order to better expose the medial portion of the distal femur. A longitudinal incision is performed 1 cm proximal to the medial epicondyle, extending proximally for 10–12 cm. Careful dissection of the subcutaneous tissue is performed, and the fascia is incised longitudinally. Vastus medialis muscle is separated from the intermuscular septum and elevated by two curved retractors in order to expose the anteromedial aspect of the distal femur, and the neurovascular structures are protected inferiorly by two blunt retractors throughout the surgery to prevent injury to these vital structures.

Under fluoroscopy, two Kirschner wires are inserted from medial to lateral, with the distal one inserted just proximal to the medial femoral condyle and parallel to the joint line, with careful attention to not violate the patello‐femoral joint. On the other hand, the second wire was inserted proximally with an angle matching the planned correction. The osteotomy was then performed with an oscillating saw and completed with an osteotome, carefully paying attention not to break the osteotomy hinge, and the bone wedge was extracted. After the removal of the K wires, the osteotomy was closed, carefully applying lateral pressure while stabilizing the knee joint and stabilized with an angled blade plate until 2012 and then with an angular‐stable plate and screws (TomoFix; DePuy Synthes) (Figure 1). A final fluoroscopic control is performed to verify the position of the screws and the plate and limb alignment, which is also clinically assessed at the end of the procedures.

**Figure 1:**
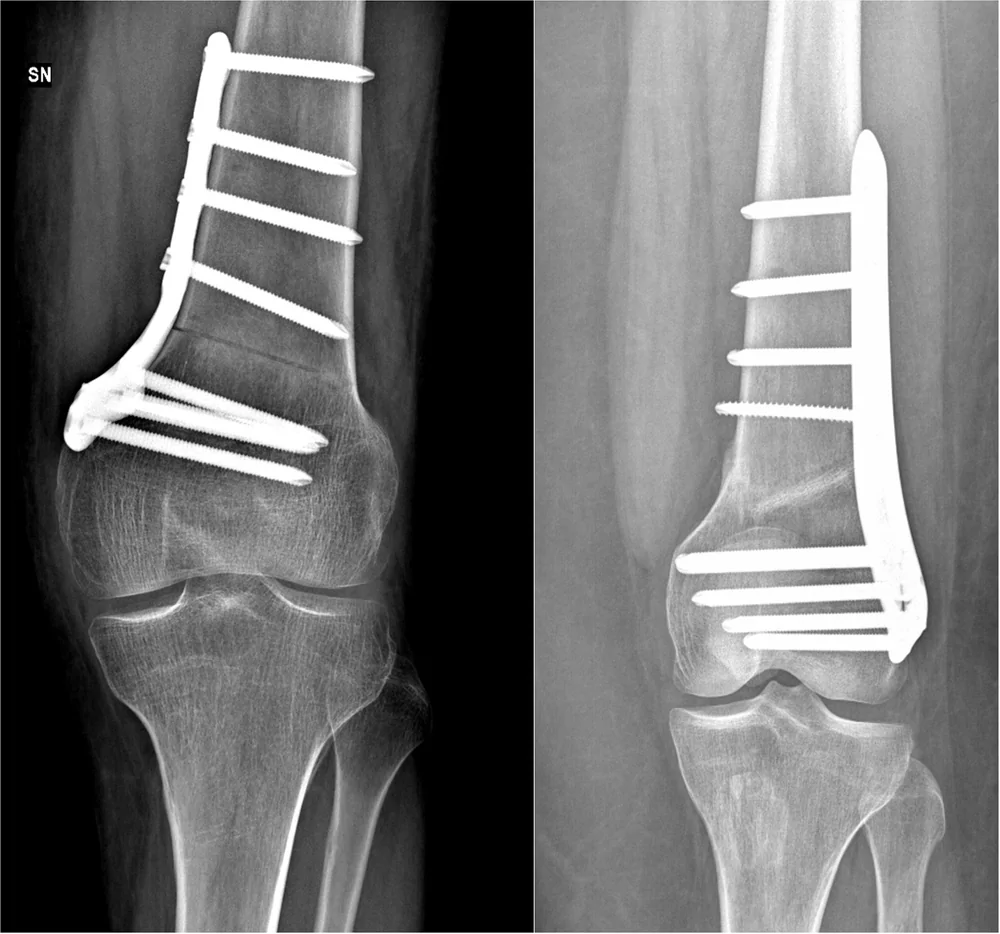
Postoperative X‐rays of a lateral opening wedge distal femoral osteotomy (right) and a closing wedge distal femoral osteotomy (left).

#### Lateral opening wedge distal femoral osteotomy (LOW‐DFO)

The surgical technique has already been described by Sherman et al. [31] (Figure 1).

A 10–15 cm longitudinal skin incision was performed on the lateral distal aspect of the femur, approximately 2 cm above the posterior border of the iliotibial band and at the level of the lateral epicondyle. The iliotibial tract is identified and split. The lateral portion of the distal femur is then exposed by dissecting the intermuscular septum and retracting the vastus lateralis anteriorly with two curved retractors, and the posterior neurovascular structures are protected by two blunt retractors.

Under fluoroscopic guidance, one guide wire is inserted from proximal‐lateral to distal‐medial, starting around 3–4 cm from the lateral epicondyle and directed towards the medial epicondyle, with care in remaining proximal to the patella‐femoral joint. Osteotomy is then performed with an oscillating saw guided by the wire and completed with osteotomes, with attention to maintaining a medial cortical hinge.

The bone gap was then gradually widened using chisels, taking care to avoid fractures until the desired correction was obtained. The leg axis was evaluated clinically and radiologically.

Once the correction was achieved, the osteotomy was secured using a plate and screws. Since 2012, an angled blade plate was utilized, and then an angular stable plate and screws (TomoFix; DePuy Synthes).

In all cases, the osteotomy gap was filled with an allograft bone wedge from a femoral head or a previously prepared wedge from the tissue bank.

### Rehabilitation protocol

The same rehabilitation protocol was prescribed to all the patients.

#### Weight‐bearing

Patients were instructed to perform toe‐touch weight bearing during the first 3 weeks, and during the fourth week, the patients started partial weight bearing (10%–20% of body weight) on the operated leg [3]. Moreover, during the first 4 weeks, an extension brace was recommended during walking and sleeping. One month after surgery, a clinical‐radiographic follow‐up was performed in order to check the healing status of the wounds, the healing progression of the osteotomy and the range of motion status. If no consolidation delay was present at the time of the visit, usually partial weight‐bearing (50% of body weight) was allowed with the aim of reaching full weight‐bearing 6 weeks after surgery. Antithromboembolic prophylaxis with low‐molecular‐weight (LMW) heparin was performed until full weight‐bearing.

#### Range of motion

The recovery of the knee range of motion began 7 days after surgery. Active and passive (with a motorized device) exercises were encouraged until complete ROM was obtained, indicatively 6 weeks after surgery.

#### Muscle strengthening

Isometric strengthening exercises were encouraged the day after surgery. At 3–4 weeks, cyclette and closed kinetic chain exercises were allowed.

### Clinical follow‐up

A clinical‐radiographic follow‐up was performed at 1, 3, 6 and 12 months after surgery; if no consolidation delays were present, the patient was allowed to resume sport‐specific exercises at 3 months.

### Statistical analysis

The HKA was presented as follows: HKA = 180° means neutral alignment, HKA > 180° means valgus deformity, HKA < 180° means varus deformity [15].

Continuous variables were reported as mean ± standard deviation, while categorical variables were expressed as frequencies and percentages.

The distribution of quantitative variables was assessed using the Shapiro–Wilk test for normality.

To assess RTS and the level at which it occurred following surgery, a contingency table analysis was performed, taking into account both the presence or absence of RTS and the level achieved compared to the pre‐symptom period. Fisher's exact test was used to evaluate differences in RTW rates.

Two separate multivariate logistic regression analyses were performed: One for absolute RTS (yes/no) and another for the question ‘Do you feel at least at the same level as before surgery?’ (yes/no) as the dependent variables. This latter variable was created by categorizing participants who returned to the same or a higher level of activity as Group Yes, and those who returned at a lower level or did not return as Group No. Independent variables included sex, body mass index, age, HKA angle, Tegner scale, Lysholm score and VAS. The multivariate analysis was applied only to variables that were significant at the univariate level (*p* < 0.1).

A *p* value < 0.05 was considered indicative of statistical significance. All statistical analyses were performed using JASP (Version 0.95.1.0; JASP Team, University of Amsterdam).

## RESULTS

A total of 46 patients met the inclusion criteria; 34 were included in the analysis of the results due to refusal or impossibility to contact the other patients (see Figure 2 for details).

**Figure 2:**
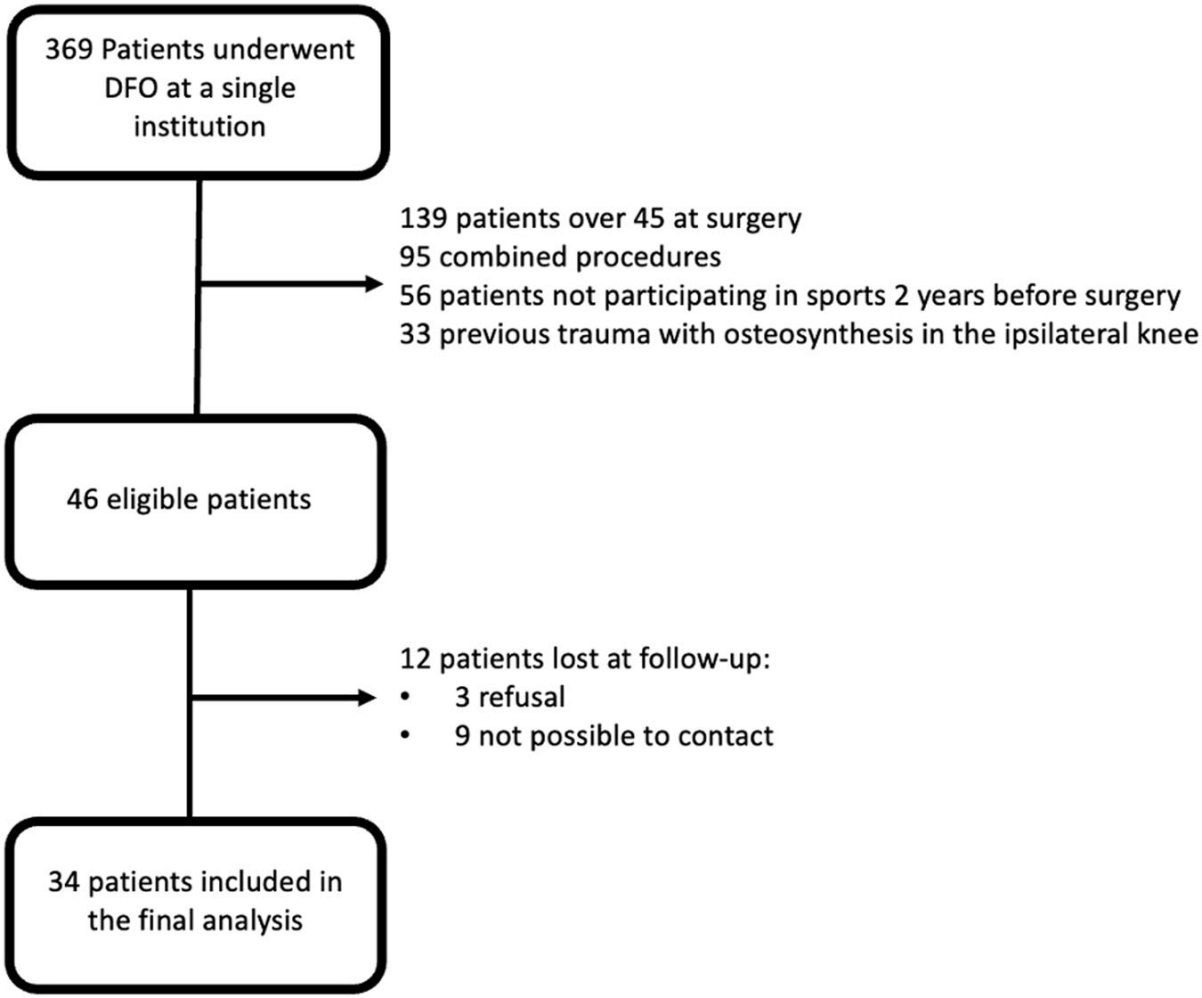
CONSORT (Consolidated Standards of Reporting Trials) diagram. DFO, distal femoral osteotomy.

The study population had an average age at surgery of 31.3 ± 9.7 years and was composed of 19 females (55.9%) and 15 males (44.1%). The surgical procedure mostly performed was MCW‐DFO (28, 82.4%). The average follow‐up time at the time of results' analyses was 7.2 ± 3.8 years.

The mean pre‐surgery HKA was 187.1 ± 3.1°, while the mean postoperative HKA was 178.1 ± 2.1°. Moreover, seven diagnostic arthroscopies (20.6%) for suspected meniscal lesions on MRI were performed, of which five did not reveal any meniscal lesion, and two required a selective lateral meniscectomy. The full demographics of our cohort are reported in detail in Table 2.

**Table 2:** Data were reported as mean ± standard deviation or *n* (%).

Demographics	
	34 patients
Age at surgery, years	31.3 ± 9.6
Body mass index, kg/m^2^	27.7 ± 5.1
Sex, *n* (%)	
Male	15 (44.1%)
Female	19 (55.9%)
Mean correction value, degrees	7.7 ± 2.5
Follow‐up time, years	7.2 ± 3.8
Follow‐up time, months	92.9 ± 44.7
Time to RTS, months	5.4 ± 5.4
Time to RTW, months	4.4 ± 2.6
Complications	4 (11.8%)

Abbreviations: RTS, return to sport; RTW, return to work.

Compared with the pre‐symptom or preoperative period, there was a significant improvement in the VAS scale (*p* < 0.001) and in the Lysholm score (*p* < 0.001). Tegner activity scale remained unchanged (*p* < 0.001). Tampa scale averaged 21.1 ± 6.9, median patient satisfaction on the Likert scale was 1 [1–5] and Subjective Patient Outcome for Return to Sports (SPORTS) score was 9 [0–10] (Table 3).

**Table 3:** PROMs collected before symptoms, before surgery and after return to sport.

PROMs				
	Before symptoms	Preoperative	Follow‐up	*p* value
Tegner scale	5.0 [3.0; 9.0]		5.0 [2.0; 8.0]	**<0.001**
Lysholm score		68.9 ± 17.2	84.7 ± 13.5	**<0.001**
VAS		5.6 ± 2.9	2.2 ± 2.3	**<0.001**
SPORTS score			9.0 [0.0; 10.0]	
Tampa scale			21.1 ± 6.9	
Likert scale			1.0 [1.0; 5.0]	

*Note*: Tegner, Likert scales and SPORTS score are presented as median and range (Mann–Whitney *U* test was used for statistical comparison); the other variables are presented as mean and standard deviation (Student's *t* test was used for statistical comparison). Bold values denote statistical significane (*p* < 0.05).

Abbreviations: PROMS, patient‐reported outcomes measurements; SPORTS, Subjective Patient Outcome for Return to Sports; VAS, visual analogue scale.

### RTS

The RTS rate in the study cohort was 79.4% (27/34) (Table 4). Of these, 85.2% (23/27) maintained the same impact activity level, while 14.8% (4/27) resumed sports activity at a lower impact level.

**Table 4:** Return to sport data, impact activity level between before symptoms and at maximum level after surgery, subjective level of sport activity between before symptoms and at maximum level after surgery.

Return to sport		
	Yes	No
Return to sport	27 (79.4)	7 (20.6)
Impact level?		
Same	23 (85.2)	—
Higher	0 (0.0)	
Lower	4 (14.8)	
Do you feel at least at the same level as before symptoms?		
Same	12 (44.5)	—
Higher	7 (25.9)	
Lower	8 (29.6)	
Sport participation at follow‐up	23 (67.7)	11 (32.3)

*Note*: Data were presented as *n* (%).

At the final follow‐up, 67.7% (23/34) of patients reported continued participation in sports with a mean of 3.5 ± 2.9 h weekly. Over two‐thirds of the patients who returned to sport activity after surgery (70.4%, 19/27) reported subjectively maintaining or exceeding their pre‐symptom level (Table 4).

Among the 34 participants, 26.5% (9/34) engaged in high‐impact sports, 44.1% (15/34) in intermediate‐impact sports and 29.4% (10/34) in low‐impact sports during the pre‐symptomatic period. Postoperatively, 11.8% (4/34) remained in the high‐impact sports, 35.3% (12/34) in the intermediate‐impact sports and 32.4% (11/34) in the low‐impact sports (for specific data, see Table 5).

**Table 5:** Impact activity level before the onset of symptoms and maximum level after surgery, divided into high, intermediate and low according to the IMPACT Activity Level Questionnaire, see Supporting Information S1: Appendix 1.

Impact activity levels of the patients before symptoms and after return to sport				
Before symptoms	Return to sport			
	High	Intermediate	Low	No sport
High (*n* = 9)	4 (11.8)	1 (2.9)	3 (8.8)	1 (2.9)
Intermediate (*n* = 15)	0 (0.0)	11 (32.3)	0 (0.0)	4 (11.8)
Low (*n* = 10)	0 (0.0)	0 (0.0)	8 (23.5)	2 (5.9)
Total (*n* = 34)	4 (11.8)	12 (35.3)	11 (32.3)	7 (20.6)

Regarding the analysis of RTS for single sports, a total of 23/34 (67.6%) were able to return to the same sport postoperatively. It is remarkable that among patients who were playing soccer before the onset of symptoms, only two out of the five patients resumed soccer after surgery (40%); however, four‐fifths returned to play any sport among patients who were playing soccer before the onset of symptoms (80%). For full data regarding RTS in single sports, see Table 6.

**Table 6:** RTS rate divided by single sport in our cohort.

Pre‐symptoms sport	No. of patients	RTS rate (same sport)	RTS rate (including all sports)
Soccer	5	2/5 (40%)	4/5 (80%)
Swimming	7	6/7 (85.7%)	6/7 (85.7%)
Gym/Weightlifting	10	6/10 (60%)	6/10 (60%)
Dance	3	2/3 (66.7%)	2/3 (66.7%)
Other sports	9	7/9 (77.8%)	9/9 (100%)

*Note*: Other sports line includes: skiing, volleyball, fencing, karate and rugby.

Abbreviation: RTS, return to sport.

### Factors influencing RTS

Univariate regression analysis showed that the pre‐symptom Tegner scale was significant predictor of RTS, with higher pre‐symptom Tegner associated with odds ratio (OR) = 2.33 (*p* = 0.018, Table 7).

**Table 7:** Univariate regression analysis with return to sport as a variable.

Univariate logistic regression for return to sport				
	Return to sport (*n* = 27)	No return to sport (*n* = 7)	OR [95% CI]	*p* value
Age, years	30.4 ± 10.3	35.0 ± 4.7	0.95 [0.86; 1.04]	0.235
Sex (M/F)	13/14	2/5	2.32 [0.38; 14.12]	0.344
BMI	27.2 ± 5.3	29.9 ± 3.4	0.90 [0.75; 1.07]	0.202
HKA	187.1 ± 3.3	187.0 ± 2.5	1.02 [0.76; 1.36]	0.907
Tegner scale	5.0 [3.0; 9.0]	4.0 [3.0; 7.0]	2.33 [0.923; 5.85]	**0.018**
Lysholm score	68.5 ± 15.1	70.6 ± 24.9	0.99 [0.94; 1.05]	0.771
VAS	5.7 ± 2.8	5.3 ± 3.5	1.05 [0.79; 1.40]	0.753

*Note*: Data were presented as median [min; max] or mean ± SD. Asterisk represented a statistically significant association. Bold values denote statistical significance (*p* < 0.05).

Abbreviations: BMI, body mass index; CI, confidence interval; HKA, hip–knee–ankle; OR, odds ratio; SD, standard deviation; VAS, visual analogue scale.

For the subjective outcome ‘Do you feel at least at the same level as before surgery?’, none of the preoperative variables analysed emerged as a significant predictor (Supporting Information S1: Appendix 2).

### RTW

All the patients returned to work at a mean of 4.4 ± 2.6 months. Changes in occupational activity levels before and after surgery are summarized in Supporting Information S1: Appendix 3. Among patients who had a sedentary job prior to surgery, eight remained in sedentary roles, while four transitioned to an active occupation.

In contrast, of the patients who were actively employed before surgery, 19 continued in active job roles postoperatively, whereas 3 shifted to sedentary work.

Fisher's exact test confirmed that the association between baseline and post‐intervention job activity levels was statistically significant (*p* = 0.005).

### Complications and reoperations

Three major complications were registered in our cohort of patients (8.8%) requiring further hospital admission: one intraoperative fracture, one postoperative deep infection treated with antibiotics and implant retention in line with European Society of Sports Traumatology, Knee Surgery and Arthroscopy (ESSKA) guidelines [27] and one postoperative deep venous thrombosis. Moreover, one minor complication (5%) was registered, consisting of residual postoperative stiffness that required mobilization under anaesthesia.

During the follow‐up period, a total of 13 (38.2%) reoperations were performed: 12/34 (35.3%) plate removals for symptomatic hardware and one arthroscopy for osteophyte removal and patellar chondroplasty.

No revisions of osteotomy or conversion to unicompartmental knee arthroplasty or total knee arthroplasty were registered in our cohort; hence, the survival rate of this procedure at the time of follow‐up was 100%.

## DISCUSSION

The most important finding of the present study is that 8 out of 10 young and active patients who underwent DFO were able to RTS after a period of less than 6 months, and that two out of three patients are still active after a mean follow‐up of 7 years.

The literature data on RTS on DFO are limited, as a recent systematic review found only five studies investigating this procedure [9]. In this review, including 76 patients, with the vast majority of them (92%) undergoing lateral OW procedure, the RTS rate was reported as 86% at a mean follow‐up time of 1 year. However, the indications of this surgical procedure were different among the studies, and in some of those studies, the indication for DFO was the treatment of lateral compartment knee OA. Moreover, a non‐negligible amount of combined procedures, such as lateral meniscus transplant and several cartilage treatments, were performed as well among those different studies [7, 28, 34].

In a study with a similar patient demographics and follow‐up time, investigating outcomes of isolated DFO, including 17 patients, reported a 70% RTS rate with a mean time of 9.5 months [1]. Compared to the present study, the increased time to RTS may be related to the possible slower healing and bone allograft integration after the lateral OW procedure that has been reported to be completed at about 6 months after surgery [6]. Similarly, a slightly longer time to RTS, 11 months on average, was reported by Voleti et al. in a retrospective series of 13 patients after isolated DFO [36]. As highlighted by a systematic review, there is high variability of RTS time, with the mean time reported to be 12 months [9]. This is another key aspect because when DFO is performed with the aim of returning to sport, the rehabilitation, especially the part focused on proprioception and sport‐specific tasks, may require a longer time compared to the mere bone healing [13].

When evaluating the RTS of a surgical procedure, attention must be turned to the postoperative activity modifications that are frequently observed after surgery. In the present study, two out of three patients resumed the same sport, while among the patients who returned to sport, 85.2% (23/27) maintained the same impact activity level, 14.8% (4/27) resumed sports activity at a lower level, and no one was able to increase the impact level. This information is a key message as it helps to better set the expectation of this procedure when performed in active individuals with a valgus knee who wish to continue or improve their sports performance. In fact, while the procedure could guarantee some level of sport participation in the vast majority of patients, it is unable to improve the impact level of the sports practised.

The sport‐specific data of the present study are limited by the small number of patients within each subgroup. Nevertheless, this study is among the first to report return to soccer following DFO, which was achieved by only 40% of patients (two of five). These findings are consistent with previously reported inferior outcomes in soccer players, as other studies showed that only four of seven patients (57%) were able to return to soccer after DFO, with just one patient (14%) remaining active at a mean follow‐up of 3 years. Overall, soccer appears to be associated with less favourable RTS outcomes after knee realignment procedures, likely due to the high rotational and impact demands of the sport [8, 16].

In the present study, 13 patients (38.2%) required additional surgery, ranging from arthroscopy to hardware removal or early complications. Although this reoperation rate is consistent with those reported in similar studies, and despite overall satisfactory clinical outcomes, patients should be counselled preoperatively about the relatively high likelihood of requiring further surgical procedures after DFO [9].

Resumption of sports activity after knee realignment procedures, particularly for high‐impact sports, is influenced by a multitude of factors [34]. While several predictors of RTS have been described after HTO, including preoperative sports participation [17], the degree of tibial correction [19], patient motivation and low levels of kinesiophobia [21], data regarding DFO remain limited. In the present study, a higher pre‐symptom Tegner score was the only significant predictor of RTS, reinforcing the concept that patients engaged in higher levels of sports activity before symptom onset may present with better baseline physical condition and greater motivation to resume physical activity. Moreover, although not specifically investigated in the current literature, the inclusion of sport as a meaningful component of a patient's daily activities and personal goals is likely to play an important role in the RTS process and should be considered during preoperative counselling and patient selection.

The results of the present study should be interpreted in light of several limitations. First, the retrospective design of the study may have introduced selection bias. Second, the relatively small sample size limits the statistical power of the analysis. However, it should be noted that the strict inclusion criteria (isolated DFO and patients younger than 45 years) were adopted to provide focused data on a specific and understudied patient subgroup. Third, the lack of imaging evaluation at final follow‐up prevented direct assessment of the influence of additional factors, such as progression of OA in the lateral and medial compartments, which may have affected RTS outcomes. Fourth, the preoperative and the pre‐symptoms patient‐reported outcome measures were collected at follow‐up, leading to potential recall biases. Finally, patients included in the cohort underwent different surgical techniques, which may represent a further source of heterogeneity.

Despite these limitations, the present study provides clinically meaningful information for the management of young and physically active patients undergoing DFO. Specifically, the identification of pre‐symptom activity level as the main predictor of RTS may assist surgeons in preoperative counselling, patient selection, and expectation management. These findings support the concept that DFO can represent a viable joint‐preserving option in selected young patients with valgus malalignment, particularly in those who are highly active and motivated to resume sports participation.

## CONCLUSIONS

DFO performed in a young and active population results in high RTS rates and continuous sports participation even 7 years after surgery. Moreover, higher pre‐symptoms Tegner scale was associated with higher odds of returning to sport compared to lower Tegner scale values.

## AUTHOR CONTRIBUTIONS

Gian Andrea Lucidi, Alberto Grassi and Stefano Zaffagnini had the initial idea of the study and supervised the work by reviewing the paper and conducting the decision‐making processes. Federico Maria Adravanti, Claudio Rossi, Margherita Bonaiuti and Stefano Di Paolo contributed to Material preparation, data collection and analysis. Gian Andrea Lucidi, Federico Maria Adravanti and Claudio Rossi contributed to the writing of the first draft of the manuscript.

## CONFLICT OF INTEREST STATEMENT

Stefano Zaffagnini reports a relationship with Smith & Nephew Inc and Johnson & Johnson MedTech that includes consulting or advisory and is the editor‐in‐chief of the *Journal of Experimental Orthopedics* (*JEO*). Alberto Grassi reports a relationship with Smith & Nephew Inc and Johnson & Johnson MedTech that includes consulting or advisory. The remaining authors declare no conflicts of interest.

## ETHICS STATEMENT

Approval was obtained from the ethics committee of AVEC (Area Vasta Emilia Centro) with approval no. 887/2022/Oss/IOR. The procedures used in this study adhere to the tenets of the Declaration of Helsinki. Informed consent was obtained from all individual participants and parents of participants in case of minors included in the study.

## Supporting information

Supplementary Information

## Data Availability

The data that support the findings of this study are available from the corresponding author upon reasonable request.
