# Supplementary material for: Sport participation after isolated distal femoral osteotomy in young patients: High return‐to‐sport rates and predictive role of preoperative activity level
Source: Knee Surg Sports Traumatol Arthrosc. 2026 Jul 13;34(11):4145–55. doi: 10.1002/ksa.70524 (PMC13703904; doi:10.1002/ksa.70524)
Supplement: Supplementary file 1 [file KSA-34-4145-s001.docx]

**Appendix**

| *Impact Activity Level* | | |
| --- | --- | --- |
| *High Impact* | ***Intermediate Impact*** | ***Low-Impact*** |
| Jogging | Inline Skating | Nordic Walking |
| Ice Hockey | Hiking | Cycling |
| Tennis (singles) | Mountain climbing | Bike racing |
| Squash | Mountain Biking | Swimming |
| Soccer | Fitness/ Weight-training | Water Aerobics |
| Handball | Aerobics | Cross-country skiing |
| Volleyball | Gymnastics | Golf |
| Martial arts | Downhill Skiing | Table tennis |
| Water Skiing | Snowboarding | Dancing |
| Basketball | Ice Skating | Sailing |
| Rugby | Tennis (double) | Rowing |

**Appendix 1**: Impact activity level of sports activities according to Vail et al.[1].

| ***Appendix 2 – Univariate logistic regression for RTS at the same and higher or lower level and no return*** | | | | |
| --- | --- | --- | --- | --- |
|  | ***Do you feel at least at the same level as before surgery?*** | | ***OR [95% CI]*** | ***P-Value*** |
|  | ***Yes (n=18)*** | ***No (n=16)*** |  |  |
| *Age, y* | 31.6 ± 9.3 | 31.0 ± 10.2 | 1.01 [0.94; 1.08] | **0.850** |
| *Sex (M/F)* | 8/10 | 7/9 | 1.03 [0.27; 3.99] | **0.968** |
| *BMI* | 26.2 ± 4.9 | 29.4 ± 4.8 | 0.88 [0.75; 1.02] | **0.062** |
| *HKA* | 187.0 ± 2.8 | 187.2 ± 3.5 | 0.97 [0.77; 1.23] | **0.812** |
| *Tegner scale* | 5.0 [4.0; 8.0] | 5.5 [3.0; 9.0] | 0.79 [0.53; 1.18] | **0.237** |
| *Lysholm score* | 66.3 ± 14.6 | 71.9 ± 19.7 | 0.98 [0.94; 1.02] | **0.333** |
| *VAS* | 5.3 ± 2.4 | 5.9 ± 3.4 | 0.93 [0.74; 1.19] | **0.578** |

**Appendix 2:** Univariate regression analysis with subjective level of sports activity as a variable. Data were presented as median [min; max] or mean ± SD. OR = Odds ratio; BMI = Body mass index; VAS = visual analog scale. Asterisk represented a statistically significant association.

| ***Appendix 3 – Job activity*** | | |
| --- | --- | --- |
| ***Before Surgery*** | ***After Surgery*** | |
|  | *Sedentary* | *Active* |
| *Sedentary (n = 12)* | 8 (23.5) | 4 (11.8) |
| *Active (n = 22)* | 3 (8.8) | 19 (55.9) |
| *Total (n = 34)* | 11 (32.3) | 23 (67.7) |

**Appendix 3:** Job activity of patients between pre- and postoperative period. Data were presented as n (%).

**Bibliography**

1.	Vail TP, Mallon WJ, Liebelt RA (1996) Athletic Activities After Joint Arthroplasty. Sports Medicine and Arthroscopy Review 4(3):298
